# Supplementary material for: The path toward equal performance in medical machine learning
Source: Patterns (N Y). 2023 Jul 14;4(7):100790. doi: 10.1016/j.patter.2023.100790 (PMC10382979; doi:10.1016/j.patter.2023.100790)
Supplement: Document S1. Supplemental notes [file mmc1.pdf]

Patterns, Volume *4*

Supplemental information

The path toward equal performance in medical machine learning

Eike Petersen, Sune Holm, Melanie Ganz, and Aasa Feragen

Supplemental note on the bias-variance decomposition provided in the main paper

Our setting in this paper differs from the one in Chen et al. [1] in two ways:

1. We explicitly consider a setting with label errors, i.e., the observed labels Y_{obs} may differ from the true labels Y. As we will see below, this turns out to not affect the decomposition in any way.
2. For simplicity of presentation, we here only consider the case in which the expected mean squared prediction error (or Brier score) is used as the loss function, whereas both Chen et al. [1] and Domingos [2] consider a more general setting with different loss functions. As we point out below, the decomposition provided by Chen et al. [1] fully generalizes to the setting with label errors.

The decomposition we present in the main text follows directly from Theorem 1 in Chen et al. [1]. To see this, note that Chen et al. [1] (and Domingos [2], upon which the derivation in Chen et al. is based) make no assumptions about the *process* by which the model predictions $\hat{y}_D = h_D(x, g)$ are obtained from a training set D. In fact, Chen et al. [1] simply assume that an "algorithm that learns models $\hat{Y}_D$ from datasets D is given, and the covariates X and size of the training data are fixed." They furthermore assume that "$\hat{Y}_D$ is a deterministic function $\hat{y}_D(x, a)$ given the training set D". These assumptions are fully compatible with the case in which the presence of label errors affects the learned model, and the general decomposition provided by theorem 1 in Chen et al. [1] still holds. (In fact, it is not even required that the learning mechanism takes the labels into account at all.) Applying the appropriate simplifications for the case of the squared error loss yields our decomposition. Our decomposition also follows directly from the (simpler, since less general) derivation of the mean-squared error decomposition provided by Bishop [3], by the same argument: the mechanism by which the prediction model is obtained from the training dataset is irrelevant to the derivation. Conditioning on group membership G also does not affect the derivation in any way, since datasets D are drawn independently of G. We discuss in the main text how learning from biased or noisy labels may affect the different terms of the decomposition.

Supplemental References

[1] I. Chen, F. D. Johansson, D. Sontag, Why is my classifier discriminatory?, in: S. Bengio, H. Wallach, H. Larochelle, K. Grauman, N. Cesa-Bianchi, R. Garnett (Eds.), Advances in Neural Information Processing Systems, volume 31, 2018. URL: https://proceedings.neurips.cc/paper_files/paper/2018/file/1f1baa5b8edac74eb4eaa329f14a0361-Paper.pdf.

[2] P. Domingos, A unified bias-variance decomposition and its applications, in: Proceedings of the Seventeenth International Conference on Machine Learning, ICML '00, Morgan Kaufmann Publishers Inc., San Francisco, CA, USA, 2000, p. 231–238.

[3] C. M. Bishop, Pattern Recognition and Machine Learning, Information Science and Statistics, Springer New York, NY, 2006.
