# Supplementary material for: The path toward equal performance in medical machine learning
Source: Patterns (N Y). 2023 Jul 14;4(7):100790. doi: 10.1016/j.patter.2023.100790 (PMC10382979; doi:10.1016/j.patter.2023.100790)
Supplement: Document S2. Article plus supplemental information [file mmc2.pdf]

Patterns

CellPress
OPEN ACCESS

Perspective

The path toward equal performance in medical machine learning

Eike Petersen,[1,2,*] Sune Holm,[2,3] Melanie Ganz,[2,4,5] and Aasa Feragen[1,2]
[1]DTU Compute, Technical University of Denmark, Richard Pedersens Plads, 2800 Kgs. Lyngby, Denmark
[2]Pioneer Centre for AI, Øster Voldgade 3, 1350 Copenhagen, Denmark
[3]Department of Food and Resource Economics, University of Copenhagen, Rolighedsvej 23, 1958 Frederiksberg C., Denmark
[4]Department of Computer Science, University of Copenhagen, Universitetsparken 1, 2100 Copenhagen, Denmark
[5]Neurobiology Research Unit, Rigshospitalet, Inge Lehmanns Vej 6–8, 2100 Copenhagen, Denmark
*Correspondence: ewipe@dtu.dk
https://doi.org/10.1016/j.patter.2023.100790

THE BIGGER PICTURE Machine learning models are poised to be widely used in medical applications. This raises important fairness considerations, since it has been observed that such models often are better at predicting outcomes or diagnosing diseases in some patient groups compared with others. Here, we ask: why do such performance differences occur, and what would it take to build models that perform equally well for all patients? We point out that different factors can lead to underperformance in patient groups, including underrepresentation in the training dataset, technical modeling choices, and differences in the difficulty of the prediction task between groups. In addition, biased data collection may confound both learning and performance evaluation. Narrow algorithmic fairness solutions cannot address all of these issues, and we find that leveling *up* model performance may require not only *more* data from underperforming groups but also *better* data.

1 2 3 4 5 **Concept:** Basic principles of a new data science output observed and reported

SUMMARY

To ensure equitable quality of care, differences in machine learning model performance between patient groups must be addressed. Here, we argue that two separate mechanisms can cause performance differences between groups. First, model performance may be worse than theoretically achievable in a given group. This can occur due to a combination of group underrepresentation, modeling choices, and the characteristics of the prediction task at hand. We examine scenarios in which underrepresentation leads to underperformance, scenarios in which it does not, and the differences between them. Second, the optimal achievable performance may also differ between groups due to differences in the intrinsic difficulty of the prediction task. We discuss several possible causes of such differences in task difficulty. In addition, challenges such as label biases and selection biases may confound both learning and performance evaluation. We highlight consequences for the path toward equal performance, and we emphasize that leveling *up* model performance may require gathering not only *more* data from underperforming groups but also *better* data. Throughout, we ground our discussion in real-world medical phenomena and case studies while also referencing relevant statistical theory.

INTRODUCTION

The fairness of machine learning models has come under increased scrutiny in recent years, with performance disparities between different groups being one potential source of unfairness.[1] The discussion has also reached the medical machine learning community,[2–5] where the effects of group underrepresentation have received much attention. In a recent study, Larrazabal et al.[3] found improved discriminative performance of chest X-ray-based thoracic disease classifiers for a given (gender-based) group if that group was more strongly represented in the training data. Puyol-Antón et al.[6] and Lee et al.[7] have observed similar effects of racial representation on the performance of cardiac magnetic resonance imaging (MRI) segmentation models. In a parallel development, the medical community is increasingly recognizing the harms caused by medical research focusing primarily on male and Western individuals.[8,9] Together, these developments have incited a commendable movement toward using diverse, representative datasets in medical machine learning research. However, as we emphasize here (and as has

This is an open access article under the CC BY license (http://creativecommons.org/licenses/by/4.0/).

been pointed out before), the relationship between a group's representation in the training dataset and the model performance for that group is complex. Using similar amounts of data from different groups does not ensure equal model performance across groups, and group underrepresentation does not necessarily result in poor model performance.

Consider the study by Larrazabal et al.[3] on chest X-ray-based thoracic disease classifiers. In this study, even when training a model *only* on women, the model's performance in women was still worse than in men for some diseases, such as pleural thickening and pneumothorax. The performance disparity for these diseases was even greater in the case of a balanced dataset consisting of 50% women and 50% men. Notably, a clear case of the opposite pattern (worse performance in men) was not observed for any disease. Similar observations were made by Lee et al.[7] in the context of cardiac MRI segmentation. These results illustrate that a given estimation task may be *intrinsically harder* in certain groups compared with others. In statistical terms, the mutual information, or statistical dependence, between model inputs and outputs may differ between groups.

Clear trends relating relative group representation to relative model performance are also not always observed. For instance, Lee et al.,[7] while observing a consistent relationship between *racial* representation and model performance in such groups, do not find such a relationship between *gender* representation and model performance in those groups. Our recent study on MRI-based Alzheimer's disease (AD) classification[10] provides an even more perplexing example. In this study, we found that increasing the relative representation of women in the training dataset (while keeping the total dataset size fixed) slightly improved model performance in both women *and* men. Although the trends were not very strong, they were statistically significant. This observation was surprising, considering that one might expect model performance to decline in male test subjects as their relative representation in the training dataset is reduced.

How can we explain these seemingly contradictory observations? We argue here that two separate mechanisms may cause a model to underperform in a given group compared with others. First, the model may perform suboptimally in a group due to a combination of the group's (presumably low) representation in the training dataset, the magnitude and character of the physiological differences between the groups, the modeling choices, and the selected training procedure. By optimality, we refer here (and in the following) to the optimal model performance achievable in this group and for this estimation task, given access to infinitely many training samples and ideal modeling choices. This corresponds to the notion of Bayes optimality.[11] Second, this level of optimal achievable performance may differ between groups due to differences in the intrinsic difficulty of the estimation task, corresponding to differences in the irreducible (or Bayes) error. We will discuss several possible causes of such differences in intrinsic task difficulty and highlight consequences for the path toward (more) equal model performance.

As a final introductory note, we focus here on a model's overall discriminative performance. By this, we mean the model's ability to accurately predict the true outcome labels, as measured by, e.g., the squared prediction error (also known as the Brier score), the overall prediction accuracy, the area under the receiver-operating characteristic curve (AUROC), and other similar measures. (True and false positive rates individually are not of interest to us as they can be trivially traded off against each other.) Of course, other dimensions of model performance may be equally relevant,[5,12] but they are beyond the scope of this piece.

BACKGROUND: ESTIMATOR BIAS, VARIANCE, AND IRREDUCIBLE ERROR

We begin by introducing a theoretical framework for the following discussion, adapted from the work of Chen et al.[13] In the following, let X denote input data, G group membership, Y the unobservable true labels distributed following $p(y|x,g)$, and Y_{obs} the observed but potentially noisy or biased labels. Assume furthermore that

$$\widehat{y}_D = h_D(x,g) \qquad \text{(Equation 1)}$$

denotes a model's prediction for a given input sample (x,g), where the model is learned from a training set D consisting of observations (x,g,y_{obs}). Then, given a test sample (x,g), the expectation $\mathbb{E}_D[\widehat{Y}_D] = \mathbb{E}_D[h_D(x,g)]$ denotes the average model prediction for that test sample over draws of training sets D. Moreover, given the same test sample (x,g), the expectation $\mathbb{E}_{Y|X=x,G=g}[Y]$ denotes the Bayes optimal prediction for that sample. Adapting the unified bias-variance decomposition of Domingos,[14] Chen et al.[13] provide the following decomposition (refer to the supplemental information for additional details on this decomposition) of the expected mean squared prediction error (or Brier score) for group G over draws of random training sets D:

$$\underbrace{\mathbb{E}_{D,X|G}\left[\left(\widehat{Y}_D - Y\right)^2\right]}_{\text{Expected mean squared error}} = \underbrace{\mathbb{E}_{X|G}\left[\left(\mathbb{E}_D[\widehat{Y}_D] - \mathbb{E}_Y[Y]\right)^2\right]}_{\text{Squared estimator bias}} + \underbrace{\mathbb{E}_{D,X|G}\left[\left(\mathbb{E}_D[\widehat{Y}_D] - \widehat{Y}_D\right)^2\right]}_{\text{Estimator variance}} + \underbrace{\mathbb{E}_{X|G}\left[\left(\mathbb{E}_Y[Y] - Y\right)^2\right]}_{\text{Irreducible (Bayes) error}}. \qquad \text{(Equation 2)}$$

In Equation 2, the first term on the right-hand side quantifies the error related to the model's expected deviation from the Bayes optimal prediction. The second term quantifies the error due to model variance over repeated training set draws, and the third term captures the irreducible error that even a Bayes optimal predictor will incur because Y cannot be perfectly predicted from X and G. This last term captures what is known as "aleatoric uncertainty" (or "data uncertainty"), while the estimator variance term captures "epistemic uncertainty" (or "model uncertainty").[15,16] Label errors will affect the learned model $h_D(x,g)$ and thus influence the bias and variance terms of the decomposition, but not the irreducible error term.

We will refer to back to the three components of Equation 2 throughout the following discussion. Note that for simplicity of notation, we present the decomposition only for the case of the mean squared prediction error here, but analogous decompositions hold for other losses, including overall error rate; refer to Chen et al.[13] for details. Finally, note that Equation 1 also

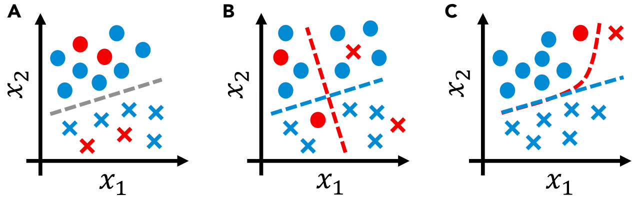

Figure 1. Illustrations of different cases of binary classification under group underrepresentation

Circles and crosses denote the two possible outcomes (values of y), blue (majority) and red (minority), two patient groups of interest. The variables x_1 and x_2 denote model inputs.

(A) Group underrepresentation is not problematic if the same decision boundary is optimal for all groups.

(B) If the optimal decision boundaries differ between groups, and either the model or the input data are not sufficiently expressive to capture the optimal decision boundaries for all groups simultaneously, standard (empirical risk minimizing) learning approaches will optimize for performance in the majority group (here, the blue group).

(C) An expressive model could learn a decision boundary (red) that is optimal for both groups. In practice, however, it is unclear whether a training procedure will indeed identify this optimal boundary. This is due to inductive biases,[19] local optimization schemes, and limited dataset size for the minority groups, all combined with standard empirical risk minimization, which prioritizes optimizing performance for the majority group.

covers the case of models that do *not* take explicit group membership into account, by simply constraining the class of permissible models $h(\cdot,\cdot)$ to those that ignore the second argument.

THE RELATIONSHIP BETWEEN GROUP REPRESENTATION AND MODEL PERFORMANCE

When *does* underrepresentation cause disproportionate reductions in model performance? This depends on several factors, including the size and composition of the dataset, the model choice, the training procedure, and the underlying estimation task. Note that, in terms of Equation 2, group underrepresentation can only ever influence the first two terms (estimator bias and variance) and never the irreducible error term.

If the inputs are sufficiently informative and the selected model class is sufficiently expressive to simultaneously reflect the optimal mapping between model inputs and outputs for all groups, being a training dataset minority does not *have* to cause suboptimal model performance (see Figures 1A and 1B). In terms of Equation 2, this corresponds to a case in which estimator bias is low—since the model is sufficiently expressive to not have to decrease performance in one group to improve performance in another—and estimator variance on the underrepresented group is also low. With highly flexible models, the latter part is the challenging one: if the model is too flexible and groups differ significantly, majority samples cannot provide helpful regularization for the minority class, and estimator variance on the minority class will be high. Thus, the existence of a mapping that is optimal for all groups simultaneously does not ensure that this optimal decision boundary is indeed learned (see Figure 1C), as can also be observed in the study of Larrazabal et al.[3] The models used in this study were highly expressive, and the patient groups under consideration (based on biological sex) could be clearly identified from chest X-ray recordings.[17,18] As has been pointed out elsewhere,[18] this enables models to internally identify patient groups and then apply different decision models to different patient groups. Thus, it would appear likely that the models considered by Larrazabal et al.[3] were capable of learning a mapping that is simultaneously optimal for women and men. Still, the authors observed significantly suboptimal model performance in the respective minority groups compared with the performance obtained when training only on that group, illustrating that optimal decision boundaries are not always learned.

Technically, the fact that training dataset minorities tend to exhibit suboptimal model performance is unsurprising. Standard deep learning approaches perform "empirical risk minimization" (ERM), which optimizes model performance over the training distribution $p_{\text{train}}(x)$. However, both the feature distribution $p(x|g)$ and the label distribution $p(y|x,g)$ may differ between patient groups g. If $p(g)$ is imbalanced with respect to different patient groups, the objective function optimized during model training is thus most strongly affected by the model's performance in the majority group. Especially in combination with inductive biases,[19] explicit regularization schemes, and the use of a local optimization method, this may prevent the training process from converging toward a mapping that is optimal not only for the majority but also for the minority groups.

The impact of differing group representation on model performance for the different groups will, of course, depend on how different the groups are, both in their input data distribution $p(x|g)$ and in their input-output mapping $p(y|x,g)$[20]. Referring back to Equation 2, the estimator bias introduced by group underrepresentation will be higher if a group differs more strongly from the majority. This might explain, for example, why we observed relatively weak trends in our previously mentioned brain MRI-based AD classification study,[10] compared with the strong trends observed by Larrazabal et al.[3] for chest X-ray-based lung disease classification: male and female chest X-ray recordings differ more strongly than male and female brain MRI scans. This is especially true once the latter are registered to a common atlas space, as we did in our study and as is standard procedure in the neuroimaging field.

Interestingly, it may even be preferable for a group to trade off its own representation against another group, as we also seem to observe in our brain MRI-based study[10]: the weak trends that we observe indicate that higher female representation at the expense of lower male representation is beneficial for both women and men. This can occur if the optimal input-output mapping is similar across groups but noisier in one group. In this case, examples from less noisy groups may prove more informative for the training process, resulting in lower estimator variance without introducing additional estimator bias.

DIFFERING MEDICAL PREDICTION TASK DIFFICULTY

Even if an optimal model is indeed learned, i.e., an input-output mapping that achieves optimal performance for all groups, there is no reason to expect the model to perform equally well for all groups. Performance disparities may still be observed due to differences in the intrinsic difficulty of the estimation problem to be solved for the different groups, corresponding to the irreducible (or Bayes) error term in Equation 2. We here define the difficulty of a medical prediction task via the Bayes optimal model performance with respect to the *true* (but typically unobservable) outcome labels Y on the target population. This differs

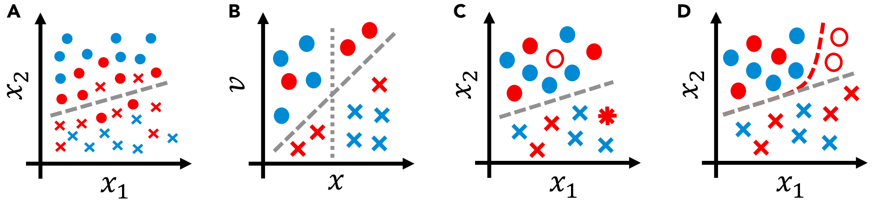

Figure 2. Illustrations of different causes of performance disparities in binary classification

Circles and crosses denote the two possible outcomes (values of y), blue and red mark two patient groups of interest. The variables x_1 and x_2 denote model inputs.

(A) Higher levels of input noise will lead to worse classification performance in the red group compared with the blue group. This might be a symptom of an unobserved cause of the outcome that is more influential in the red group than in the blue group, cf. (B).

(B) Without knowledge of the additional variable v, the blue group can be correctly classified based just on x (dotted line). This is not possible for the red group, however, which requires a decision boundary taking the additional variable v into account (dashed line).

(C) Completely random label noise will lead to worse performance metric estimates in the red group compared with the blue group, even though model performance with respect to the true labels is identical. The empty circle indicates a true circle mislabeled as a cross; the star indicates the inverse.

(D) Systematic label errors will lead to worse model performance (with respect to the true outcome labels) in the red group compared with the blue group, because a suboptimal decision boundary (red) is learned instead of the optimal one (gray). If the same systematic label errors are present in the test set, this is undetectable.

importantly from model performance with respect to the observable labels Y_{obs} on a given population, which may be subject to label noise and selection biases (see "noisy labels, sample selection biases, and misleading performance estimates"), complicating both the learning process and the performance estimation.

Differences in task difficulty have important and well-known[21,22] consequences for algorithmic approaches to fair learning that enforce, e.g., equal error rates across groups: if the optimal achievable model performance differs between groups, such approaches will actively reduce model performance for groups with lower task difficulty, an effect known as "leveling down."[23,24] This appears especially undesirable in the health-care context. Differences in prediction task difficulty may arise for several different reasons, which we will discuss below and categorize into two main groups: issues related to input disturbances and issues related to unobserved causes of the outcome.

The first group of causes of differing task difficulty concerns differing input disturbance characteristics with regard to the underlying physiological property of interest (see Figure 2A). As an example, consider chest X-ray-based disease diagnosis. Breast tissue represents a confounding occlusion in frontal chest X-ray recordings, thus corresponding to more strongly disturbed measurements of chest physiology in women.[25] This might explain why, as discussed above, Larrazabal et al.[3] found model performance for some diseases in women to be lower than in men even when using entirely female training sets. Similarly, abdominal ultrasound recordings[26] and surface electromyographic measurements[27] of obese patients are known to be of lower quality. Another potential cause of differing input disturbance levels is the disparity in the interactions of different patient groups with the medical system.[28] Such disparities could lead to the typical recording from one group being obtained using different equipment, by a different type of doctor, in a different medical environment, and at a different stage of disease, all of which may influence input disturbance characteristics.

A second important source of differences in task difficulty is given by unobserved causes of the outcome that affect outcomes more strongly in one group compared with others (see Figure 2B). As an example, fluctuations in female hormone levels represent an important factor in many diagnostic tasks,[29] whereas male hormone levels are more stable. Thus, in affected prediction tasks, task difficulty will typically be higher in women, at least when hormone level measurements are not available. As another example, the prevalence of comorbidities is often significantly higher in older cohorts compared with younger cohorts, potentially complicating prediction in older patients.

NOISY LABELS, SAMPLE SELECTION BIASES, AND MISLEADING PERFORMANCE ESTIMATES

Medical data are well known to be subject to various biases, including label biases, choice of biased proxy variables, and sample selection biases. How do such biases affect model performance in different groups?

Label noise describes the situation in which the observed labels Y_{obs} differ from the true labels Y. Label noise may be random or systematic; in the latter case, it is often called *label bias*. In the latter category, we include every type of label noise that, given a patient group, is not *noisy completely at random*.[30] This includes, for example, systematic over- or underdiagnosis. As an example of primarily *random* label noise, Zhang et al.[22] report generally high levels of label noise in standard chest X-ray datasets, with the highest noise levels being observed in the oldest patient group. Similarly, Daneshjou et al.[31] observe high levels of label noise in a dermatological dataset. Such random label noise may have two distinct effects when training a model and assessing its performance. First, the increased stochasticity of the input-output relationship may lead to reduced sample efficiency, such that more data are required to achieve the same level of model performance. Second, random label noise leads to an increase in the prediction error with respect to Y_{obs}. Importantly, however, this is not necessarily indicative of *actual* model performance being reduced, i.e., performance with respect to the true labels Y. If the label errors are truly noisy completely at random given group membership g, the correct decision boundary may still be learned.[30] Model performance with respect to the true outcome labels may then be higher than estimated on a test dataset affected by the same type of label noise (see Figure 2C). In this case, empirical performance estimates will be unreliable for assessing between-group differences in model performance.

Systematic label noise, or *label bias*, differs crucially from random label noise in that—if not addressed properly—it results in a biased decision boundary being learned (see Figure 2D). Examples of label biases in medicine abound, from gender biases in mental health diagnoses[32] to underdiagnosis of coronary microvascular dysfunction[33]—believed to primarily affect

CellPress
OPEN ACCESS

Table 1. A path forward for practitioners to help diagnose and mitigate the different causes of bias

Cause	Effect	Diagnosis	Mitigation
Label noise, label biases, selection biases	estimator bias, uninformative performance estimates	domain expertise, analyze label correlation with proxy variables,[35] gather higher-fidelity labels[22,31]	use other target variables,[31,35] bias-robust learning techniques[44–46]
Concept shift: differences in $p(y\|x)$ between groups	estimator bias	investigate effects of group balancing and model stratification[22,47]	use stratified model,[22,47] gather additional features
Low model expressivity, differences in $p(x)$ between groups	estimator bias	investigate effects of group balancing[22,47] and increasing model expressivity	increase model expressivity
Underrepresentation and highly expressive model	high estimator variance	epistemic uncertainty quantification,[15,16] analysis of sample size-performance relationship per group[13,48]	gather more samples,[48,49] decrease model expressivity, regularize
High task difficulty	high irreducible error	aleatoric uncertainty quantification,[15,16] analysis of sample size-performance relationship per group[13,48]	gather additional or alternative features,[31,50,51] reformulate prediction task or target population

While these can help diagnose and mitigate bias in practice, they do not come with guarantees, and improved diagnostics and mitigation remain an open research problem. The list of potential causes of performance differences is not exhaustive.

women—and racial biases in pain assessment.[34] Label bias can also result from a poorly chosen proxy outcome variable. A famous example of this category can be found in the study by Obermeyer et al.,[35] who analyzed a commercial clinical risk prediction algorithm. This algorithm was trained using (past) health-care costs as a proxy for health-care needs, thus neglecting disparities in access to health care between racial groups and learning a severely racially biased risk model.

Separate from issues related to label noise, *sample selection biases* may also confound both the training process and the model performance estimates.[36,37] Sample selection biases correspond to differences between the target population $p_{\text{target}}(x)$ and the population $p_{\text{train}}(x)$ from which the training set D is drawn, i.e., covariate shift. Selection biases resulting from, for example, selecting subjects based on disease status, enrollment in the health-care system, or being treated at specific hospitals, have been widely discussed in medical statistics.[38,39]

Relatedly, as has recently been pointed out,[40] *confounding factors* may affect performance estimates. If, for example, a model performs poorly in elderly subjects, and the fraction of elderly subjects is higher in the female group, then a sex-stratified performance might indicate that female subjects were disadvantaged, when in reality, it would be elderly subjects suffering from poor model performance. This is closely related to notions of ‘‘infra-marginality’’ or ‘‘intersectionality’’[41] and points toward the importance of performing fine-grained subgroup analyses.[12,42]

Label noise and selection biases raise a critical issue in the context of our discussion: the equality of (discriminative) performance metrics on a test set is neither necessary nor sufficient for fairness in terms of discriminative performance.[37,43,44] On the one hand, differences in test-set predictive performance need not be problematic if they are purely due to differing levels of completely random label noise or selection biases, while performance differences with respect to the true labels and true target distribution are less grave. On the other hand, equal discriminative performance on a test set may obscure severe predictive biases in some groups; discriminative model performance with respect to the true labels on the target population may still be highly unequal.

THE PATH FORWARD: LEVELING UP

Given what we now know about the origins of performance differences, what may be a viable path toward equal performance in medical machine learning? ‘‘Leveling down’’ by reducing performance in top-performing groups to achieve equal performance appears particularly questionable in the medical context,[24] thus ruling out many popular fairness mitigation techniques.[21–23] How can we instead level *up* performance? Table 1 provides an overview and summary of the following discussion of possible solution approaches.

We consider it essential to note that, at least in theory, it will often be possible to achieve (near) equal performance across groups without artificially reducing performance in some groups,[43,44,46,52] even across multiple performance metrics of interest simultaneously.[12,53] This may, however, require moving beyond algorithmic solutions and performing additional targeted data collection or implementing changes to clinical practice. Thus, it may not be easy to achieve. Nevertheless, in the authors' opinion, this should be the aspirational goal.

Investigating the validity of performance estimates

Before investigating any potential remedies, practitioners should assess whether observed performance differences are, indeed, *real* or whether they are a consequence of, e.g., label biases,[43,44,46] selection biases,[36–39] confounding factors,[40] or intersectional effects.[41] To address the latter, comprehensive subgroup performance analyses should be performed.[12,42] Investigating the presence or absence of label noise and label biases, however, is notoriously hard and will in almost all cases require the consultation of domain experts. Label biases, in particular, are fundamentally unobservable from purely observational data: are observed group differences due to biased labels or due to real differences? Analyzing, e.g., diagnostic biases may require conducting a dedicated and carefully planned experimental study.[31–34,54,55] Under certain mild assumptions, label biases can sometimes be assessed by investigating the relationship between the observed outcomes and alternative proxy (health) outcomes.[35] In other cases, a subset of the used data

(e.g., electronic health records) may be subjected to a more fine-grained analysis to uncover potential label errors and biases.[22] In recent years, a series of algorithmic approaches has been proposed as well, based on assumed models of the relationship between true and noisy labels and attempting to identify these relationships from the observed data.[44–46] However, the success of any such method hinges on the correctness of the modeling assumptions, which may not be satisfied in practical applications.

Addressing underrepresentation

Addressing the effects of group underrepresentation (corresponding to the estimator bias and variance terms in Equation 2) requires differentiating between three main mechanisms (also refer to Table 1). First, suppose the Bayes optimal prediction for a test sample *x* differs between groups (see Figure 1B), corresponding to concept shift between groups. This will lead to increased estimator bias in less-represented groups. In this case, the only viable solutions are either to implement group-specific predictions (corresponding to stratified model training and resulting in increased estimator variance due to reduced training set size) or to gather additional input features to resolve any differences in $p(y|x)$ between groups. Second, estimator bias in the underrepresented group may also result from low model expressivity (see Figure 1C), which should be resolved by choosing a more expressive model. Third, high model expressivity in combination with group underrepresentation may result in high estimator variance, which can be resolved only by gathering more data samples from the underrepresented group, decreasing model expressivity, or regularizing appropriately.

The problem of group underrepresentation can be framed as a "domain adaptation" or "domain generalization" problem: given data from a group-imbalanced training distribution $p_{\text{train}}(x)$, we aim to train a model that performs well on, e.g., a group-balanced target distribution $p_{\text{target}}(x)$ (thus placing equal emphasis on model performance in all groups), ideally even generalizing to previously unseen patient groups. While the former corresponds to a standard covariate shift adaptation problem[56] (a type of domain adaptation problem), the latter corresponds to asking for domain generalization.[57] These problems lie at the heart of the quest for model robustness and generalization, and proposed solution approaches abound.[57–61] So far, however, such methods have achieved only limited empirical success in mitigating the effects of underrepresentation in the medical domain[22,47] and often result in leveling down overall model performance.[22–24] Given our analysis of the root causes of performance differences, these negative results are not surprising: such methods cannot address differences in task difficulty.

What are the consequences for dataset curation? The lessons are complex, since the effects of including additional samples from a particular group on the model's performance in that group depend on a large number of factors. Promising approaches for adaptively deciding which groups to sample from have been proposed,[48,49] attempting to automatically detect harder groups during dataset construction and then sampling preferentially from those. Such approaches will prove challenging to implement in medical practice, however. For now, the most practical recommendation still appears to be the gathering of diverse and representative datasets.

Leveling up by addressing differing task difficulty

Truly leveling up performance requires addressing differences in the difficulty of the prediction task between groups, corresponding to differences in the irreducible (Bayes) error term in Equation 2. This is a task that can be solved only in close collaboration with medical experts, as it will typically require identifying or even newly developing appropriate additional (or alternative) measurement modalities that may help resolve residual uncertainty in patient groups affected by high task difficulty. Once such additional measurements have been identified, algorithmic approaches may help adaptively select patients (or patient groups) that will benefit from gathering those (potentially costly) features.[50,51] To provide a positive example from this category, Daneshjou et al.[31] were able to strongly reduce performance differences between skin colors in dermatological disease classification models by fine-tuning the models on additional data with biopsy-proven ground truth labels.

The need for improved root cause diagnosis methods

The targeted application of the mitigation techniques outlined above is feasible only if the specific cause of an observed performance disparity has been *diagnosed* in the first place. This is an area in urgent need of further research, and we can merely aim to suggest possible approaches. Estimator variance in different groups can be assessed using epistemic model uncertainty quantification methods, while aleatoric uncertainty quantification can help compare task difficulty between groups.[15,16] Chen et al.[13] and Cai et al.[48] suggest analyzing the trajectory of performance improvements in different groups as more samples are added, to identify groups that benefit the most from additional samples. Similarly, if some groups benefit from group balancing, this may indicate the presence of estimator bias due to insufficient model expressivity. However, all of these approaches rely on the correctness of the observed labels; hence, investigations of group-specific label noise and label biases are crucial.

CONCLUSION

We have argued here that there *is* a path toward equal performance in most medical applications. It may, however, be long and winding. Starting with an assessment of potential biases in the model's performance evaluation, the path will lead past an investigation of the root causes of performance differences to tailored mitigation approaches that address them. While model choice matters[59,62] and algorithmic approaches may help,[22,57,59] these cannot resolve differences in task difficulty between groups without leveling down performance. To truly level *up* performance, researchers must reconsider the setup of the estimation task and the data collection procedure. Not only *more* data may be needed to improve performance in underperforming groups, but also *different* and *better* data.[31] There is no simple relationship between a group's representation in the training dataset and model performance in that group, and performance may or may not improve when including more examples of a given group.

Crucially, none of the discussed root causes of performance differences between groups indicate that such differences are unsalvageable. However, the precise and principled

identification of the most efficient mitigation approach in a given application remains an important open problem. Finally, to be very clear, we do not wish to detract in any way from the importance of ensuring broad representation in medical datasets. Representation *is* important and *does* matter.[3,8,9,31]

SUPPLEMENTAL INFORMATION

Supplemental information can be found online at https://doi.org/10.1016/j.patter.2023.100790.

ACKNOWLEDGMENTS

Work on this project was partially funded by the Independent Research Fund Denmark (DFF grant 9131-00097B), Denmark's Pioneer Center for AI (DNRF grant P1), and the Novo Nordisk Foundation through the Center for Basic Machine Learning Research in Life Science (MLLS, grant NNF20OC0062606). The funding agencies had no influence on the writing of the manuscript nor on the decision to submit it for publication.

AUTHOR CONTRIBUTIONS

E.P., M.G., and A.F. conceptualized the manuscript. E.P. wrote the original draft of the manuscript and conceptualized and created all figures. All authors contributed to the review and editing of the manuscript. M.G., A.F., and S.H. were responsible for funding acquisition and project administration.

DECLARATION OF INTERESTS

The authors declare no competing interests.

REFERENCES

1. Buolamwini, J., and Gebru, T. (2018). Gender Shades: Intersectional Accuracy Disparities in Commercial Gender Classification. In Proceedings of the 1st Conference on Fairness, Accountability and Transparency, *81*, S.A. Friedler and C. Wilson, eds. (PMLR), pp. 77–91. http://proceedings.mlr.press/v81/buolamwini18a.html.

2. Ricci Lara, M.A., Echeveste, R., and Ferrante, E. (2022). Addressing fairness in artificial intelligence for medical imaging. Nat. Commun. *13*, 4581. https://doi.org/10.1038/s41467-022-32186-3.

3. Larrazabal, A.J., Nieto, N., Peterson, V., Milone, D.H., and Ferrante, E. (2020). Gender imbalance in medical imaging datasets produces biased classifiers for computer-aided diagnosis. Proc. Natl. Acad. Sci. USA *117*, 12592–12594. https://doi.org/10.1073/pnas.1919012117.

4. Seyyed-Kalantari, L., Liu, G., McDermott, M., Chen, I.Y., and Ghassemi, M. (2020). CheXclusion: Fairness gaps in deep chest X-ray classifiers. In Pacific Symposium on Biocomputing (World Scientific). https://doi.org/10.1142/9789811232701_0022.

5. Rajkomar, A., Hardt, M., Howell, M.D., Corrado, G., and Chin, M.H. (2018). Ensuring Fairness in Machine Learning to Advance Health Equity. Ann. Intern. Med. *169*, 866–872. https://doi.org/10.7326/m18-1990.

6. Puyol-Antón, E., Ruijsink, B., Piechnik, S.K., Neubauer, S., Petersen, S.E., Razavi, R., and King, A.P. (2021). Fairness in Cardiac MR Image Analysis: An Investigation of Bias Due to Data Imbalance in Deep Learning Based Segmentation. In Medical Image Computing and Computer Assisted Intervention – MICCAI 2021 (Springer International Publishing), pp. 413–423. https://doi.org/10.1007/978-3-030-87199-4_39.

7. Lee, T., Puyol-Antón, E., Ruijsink, B., Shi, M., and King, A.P. (2022). A Systematic Study of Race and Sex Bias in CNN-Based Cardiac MR Segmentation. In Statistical Atlases and Computational Models of the Heart. Regular and CMRxMotion Challenge Papers (Springer Nature Switzerland), pp. 233–244. https://doi.org/10.1007/978-3-031-23443-9_22.

8. The "All of Us" Research Program, Denny, J.C., Rutter, J.L., Goldstein, D.B., Philippakis, A., Smoller, J.W., Jenkins, G., and Dishman, E. (2019). The "All of Us" Research Program. N. Engl. J. Med. *381*, 668–676. https://doi.org/10.1056/nejmsr1809937.

9. Wenger, N.K. (2012). Women and Coronary Heart Disease: A Century After Herrick. Circulation *126*, 604–611. https://doi.org/10.1161/circulationaha.111.086892.

10. Petersen, E., Feragen, A., da Costa Zemsch, M.L., Henriksen, A., Wiese Christensen, O.E., and Ganz, M. (2022). Feature Robustness and Sex Differences in Medical Imaging: A Case Study in MRI-Based Alzheimer's Disease Detection. In Medical Image Computing and Computer Assisted Intervention – MICCAI 2022 (Springer Nature Switzerland), pp. 88–98. https://doi.org/10.1007/978-3-031-16431-6_9.

11. James, G., Witten, D., Hastie, T., and Tibshirani, R. (2021). An Introduction to Statistical Learning (Springer US). https://doi.org/10.1007/978-1-0716-1418-1.

12. Petersen, E., Ganz, M., Holm, S., and Feragen, A. (2023). On (assessing) the fairness of risk score models. Proceedings of the 2023 ACM Conference on Fairness, Accountability, and Transparency. https://doi.org/10.1145/3593013.3594045.

13. Chen, I., Johansson, F.D., and Sontag, D. (2018). Why Is My Classifier Discriminatory? In Advances in Neural Information Processing Systems, *31*, S. Bengio, H. Wallach, H. Larochelle, K. Grauman, N. Cesa-Bianchi, and R. Garnett, eds. https://proceedings.neurips.cc/paper_files/paper/2018/file/1f1baa5b8edac74eb4eaa329f14a0361-Paper.pdf.

14. Domingos, P. (2000). A Unified Bias-Variance Decomposition and Its Applications. In Proceedings of the Seventeenth International Conference on Machine Learning, ICML '00 (Morgan Kaufmann Publishers Inc.).

15. Kurz, A., Hauser, K., Mehrtens, H.A., Krieghoff-Henning, E., Hekler, A., Kather, J.N., Fröhling, S., von Kalle, C., and Brinker, T.J. (2022). Uncertainty Estimation in Medical Image Classification: Systematic Review. JMIR Med. Inform. *10*, e36427. https://doi.org/10.2196/36427.

16. Hüllermeier, E., and Waegeman, W. (2021). Aleatoric and epistemic uncertainty in machine learning: an introduction to concepts and methods. Mach. Learn. *110*, 457–506. https://doi.org/10.1007/s10994-021-05946-3.

17. Yi, P.H., Wei, J., Kim, T.K., Shin, J., Sair, H.I., Hui, F.K., Hager, G.D., and Lin, C.T. (2021). Radiology "forensics": determination of age and sex from chest radiographs using deep learning. Emerg. Radiol. *28*, 949–954. https://doi.org/10.1007/s10140-021-01953-y.

18. Glocker, B., Jones, C., Bernhardt, M., and Winzeck, S. (2023). Algorithmic encoding of protected characteristics in chest X-ray disease detection models. EBioMedicine *89*, 104467. https://doi.org/10.1016/j.ebiom.2023.104467.

19. Li, Y., and Liang, Y. (2018). Learning Overparameterized Neural Networks via Stochastic Gradient Descent on Structured Data. In Advances in Neural Information Processing Systems, *31*, S. Bengio, H. Wallach, H. Larochelle, K. Grauman, N. Cesa-Bianchi, and R. Garnett, eds. (Curran Associates, Inc.). https://proceedings.neurips.cc/paper/2018/file/54fe976ba170c19ebae453679b362263-Paper.pdf.

20. Zhang, Y., Liu, T., Long, M., and Jordan, M. (2019). Bridging Theory and Algorithm for Domain Adaptation. In Proceedings of the 36th International Conference on Machine Learning, *97*, K. Chaudhuri and R. Salakhutdinov, eds. (PMLR), pp. 7404–7413. https://proceedings.mlr.press/v97/zhang19i.html.

21. Hardt, M., Price, E., and Srebro, N. (2016). Equality of opportunity in supervised learning. In Advances in Neural Information Processing Systems, pp. 3315–3323.

22. Zhang, H., Dullerud, N., Roth, K., Oakden-Rayner, L., Pfohl, S.R., and Ghassemi, M. (2022). Improving the Fairness of Chest X-Ray Classifiers. Proceedings of the Conference on Health, Inference, and Learning (CHIL)*174* (PMLR), pp. 204–233.

23. Zietlow, D., Lohaus, M., Balakrishnan, G., Kleindessner, M., Locatello, F., Schölkopf, B., and Russell, C. (2022). Leveling Down in Computer Vision: Pareto Inefficiencies in Fair Deep Classifiers. In Proceedings of the IEEE/CVF Conference on Computer Vision and Pattern Recognition (CVPR), pp. 10410–10421.

24. Mittelstadt, B., Wachter, S., and Russell, C. (2023). The Unfairness of Fair Machine Learning: Levelling Down and Strict Egalitarianism by Default. Mich. Technology Law Rev. https://ssrn.com/abstract=4331652.

25. Alexander, C. (1958). The Elimination of Confusing Breast Shadows in Chest Radiography. Australas. Radiol. *2*, 107–108. https://doi.org/10.1111/j.1440-1673.1958.tb00882.x.

26. Brahee, D.D., Ogedegbe, C., Hassler, C., Nyirenda, T., Hazelwood, V., Morchel, H., Patel, R.S., and Feldman, J. (2013). Body Mass Index and Abdominal Ultrasound Image Quality. J. Diagn. Med. Sonogr. *29*, 66–72. https://doi.org/10.1177/8756479313476919.

27. Kuiken, T.A., Lowery, M.M., and Stoykov, N.S. (2003). The effect of subcutaneous fat on myoelectric signal amplitude and cross-talk. Prosthet. Orthot. Int. *27*, 48–54. https://doi.org/10.3109/03093640309167976.

28. Ross, A.B., Kalia, V., Chan, B.Y., and Li, G. (2020). The influence of patient race on the use of diagnostic imaging in United States emergency departments: data from the National Hospital Ambulatory Medical Care survey. BMC Health Serv. Res. *20*, 840. https://doi.org/10.1186/s12913-020-05698-1.

29. Seeman, M.V., and González-Rodríguez, A. (2021). Stratification by Sex and Hormone Level When Contrasting Men and Women in Schizophrenia Trials Will Improve Personalized Treatment. J. Pers. Med. *11*, 929. https://doi.org/10.3390/jpm11090929.

30. Frénay, B., and Verleysen, M. (2014). Classification in the Presence of Label Noise: A Survey. IEEE Trans. Neural Netw. Learn. Syst. *25*, 845–869. https://doi.org/10.1109/tnnls.2013.2292894.

31. Daneshjou, R., Vodrahalli, K., Novoa, R.A., Jenkins, M., Liang, W., Rotemberg, V., Ko, J., Swetter, S.M., Bailey, E.E., Gevaert, O., et al. (2022). Disparities in dermatology AI performance on a diverse, curated clinical image set. Sci. Adv. *8*, eabq6147. https://doi.org/10.1126/sciadv.abq6147.

32. Smith, D.T., Mouzon, D.M., and Elliott, M. (2018). Reviewing the Assumptions About Men's Mental Health: An Exploration of the Gender Binary. Am. J. Men's Health *12*, 78–89. https://doi.org/10.1177/1557988316630953.

33. Dean, J., Cruz, S.D., Mehta, P.K., and Merz, C.N.B. (2015). Coronary microvascular dysfunction: sex-specific risk, diagnosis, and therapy. Nat. Rev. Cardiol. *12*, 406–414. https://doi.org/10.1038/nrcardio.2015.72.

34. Hoffman, K.M., Trawalter, S., Axt, J.R., and Oliver, M.N. (2016). Racial bias in pain assessment and treatment recommendations, and false beliefs about biological differences between blacks and whites. Proc. Natl. Acad. Sci. USA *113*, 4296–4301. https://doi.org/10.1073/pnas.1516047113.

35. Obermeyer, Z., Powers, B., Vogeli, C., and Mullainathan, S. (2019). Dissecting racial bias in an algorithm used to manage the health of populations. Science *366*, 447–453. https://doi.org/10.1126/science.aax2342.

36. Zadrozny, B. (2004). Learning and evaluating classifiers under sample selection bias. In 21st International Conference on Machine learning - ICML '04 (ACM Press). https://doi.org/10.1145/1015330.1015425.

37. Kallus, N., and Zhou, A. (2018). Residual Unfairness in Fair Machine Learning from Prejudiced Data. In Proceedings of the 35th International Conference on Machine Learning, *80*, J. Dy and A. Krause, eds. (PMLR), pp. 2439–2448. https://proceedings.mlr.press/v80/kallus18a.html.

38. Ellenberg, J.H. (1994). Selection bias in observational and experimental studies. Stat. Med. *13*, 557–567. https://doi.org/10.1002/sim.4780130518.

39. Natanson, C., Esposito, C.J., and Banks, S.M. (1998). The sirens' songs of confirmatory sepsis trials: selection bias and sampling error. Crit. Care Med. *26*, 1927–1931.

40. Mukherjee, P., Shen, T.C., Liu, J., Mathai, T., Shafaat, O., and Summers, R.M. (2022). Confounding factors need to be accounted for in assessing bias by machine learning algorithms. Nat. Med. *28*, 1159–1160. https://doi.org/10.1038/s41591-022-01847-7.

41. Simoiu, C., Corbett-Davies, S., and Goel, S. (2017). The problem of infra-marginality in outcome tests for discrimination. Ann. Appl. Stat. *11*. https://doi.org/10.1214/17-aoas1058.

42. Wang, A., Ramaswamy, V.V., and Russakovsky, O. (2022). Towards Intersectionality in Machine Learning: Including More Identities, Handling Underrepresentation, and Performing Evaluation. In 2022 ACM Conference on Fairness, Accountability, and Transparency (ACM). https://doi.org/10.1145/3531146.3533101.

43. Wick, M., Panda, S., and Tristan, J.-B. (2019). Unlocking Fairness: a Trade-off Revisited. In Advances in Neural Information Processing Systems, *32*, H. Wallach, H. Larochelle, A. Beygelzimer, F. d'Alché-Buc, E. Fox, and R. Garnett, eds. (Curran Associates, Inc.). https://proceedings.neurips.cc/paper_files/paper/2019/file/373e4c5d8edfa8b74fd4b6791d0cf6dc-Paper.pdf.

44. Sharma, M., Deshpande, A., and Shah, R.R. (2023). On Testing and Comparing Fair classifiers under Data Bias. Preprint at arXiv. https://doi.org/10.48550/ARXIV.2302.05906.

45. Wang, J., Liu, Y., and Levy, C. (2021). Fair Classification with Group-Dependent Label Noise. In Proceedings of the 2021 ACM Conference on Fairness, Accountability, and Transparency (ACM). https://doi.org/10.1145/3442188.3445915.

46. Blum, A., and Stangl, K. (2020). Recovering from Biased Data: Can Fairness Constraints Improve Accuracy? In 1st Symposium on Foundations of Responsible Computing (FORC 2020) vol. 156 of Leibniz International Proceedings in Informatics (LIPIcs), A. Roth, ed. (Schloss Dagstuhl–Leibniz-Zentrum für Informatik). https://doi.org/10.4230/LIPIcs.FORC.2020.3 https://drops.dagstuhl.de/opus/volltexte/2020/12019.

47. Pfohl, S.R., Zhang, H., Xu, Y., Foryciarz, A., Ghassemi, M., and Shah, N.H. (2022). A comparison of approaches to improve worst-case predictive model performance over patient subpopulations. Sci. Rep. *12*, 3254. https://doi.org/10.1038/s41598-022-07167-7.

48. Cai, W., Encarnacion, R., Chern, B., Corbett-Davies, S., Bogen, M., Bergman, S., and Goel, S. (2022). Adaptive Sampling Strategies to Construct Equitable Training Datasets. In 2022 ACM Conference on Fairness, Accountability, and Transparency (ACM). https://doi.org/10.1145/3531146.3533203.

49. Shekhar, S., Fields, G., Ghavamzadeh, M., and Javidi, T. (2021). Adaptive Sampling for Minimax Fair Classification. In Advances in Neural Information Processing Systems, *34*, M. Ranzato, A. Beygelzimer, Y. Dauphin, P. Liang, and J.W. Vaughan, eds. (Curran Associates, Inc.), pp. 24535–24544. https://proceedings.neurips.cc/paper_files/paper/2021/file/cd7c230fc5deb01ff5f7b1be1acef9cf-Paper.pdf.

50. Noriega-Campero, A., Bakker, M.A., Garcia-Bulle, B., and Pentland, A. (2019). Active Fairness in Algorithmic Decision Making. In Proceedings of the 2019 AAAI/ACM Conference on AI, Ethics and Society (ACM). https://doi.org/10.1145/3306618.3314277.

51. Fong, H., Kumar, V., Mehrotra, A., and Vishnoi, N.K. (2022). Fairness for AUC via Feature Augmentation. In 2022 ACM Conference on Fairness, Accountability, and Transparency (ACM). https://doi.org/10.1145/3531146.3533126.

52. Dutta, S., Wei, D., Yueksel, H., Chen, P.-Y., Liu, S., and Varshney, K. (2020). Is There a Trade-Off Between Fairness and Accuracy? A Perspective Using Mismatched Hypothesis Testing. In Proceedings of the 37th International Conference on Machine Learning, *119*, H.D. III and A. Singh, eds. (PMLR), pp. 2803–2813. https://proceedings.mlr.press/v119/dutta20a.html.

53. Lazar Reich, C., and Vijaykumar, S. (2020). A Possibility in Algorithmic Fairness: Can Calibration and Equal Error Rates Be Reconciled? In 2nd Symposium on Foundations of Responsible Computing (FORC 2021)vol. 192 of Leibniz International Proceedings in Informatics (LIPIcs), K. Ligett and S. Gupta, eds. (Schloss Dagstuhl–Leibniz-Zentrum für Informatik). https://doi.org/10.4230/LIPIcs.FORC.2021.4 https://drops.dagstuhl.de/opus/volltexte/2021/13872.

54. FitzGerald, C., and Hurst, S. (2017). Implicit bias in healthcare professionals: a systematic review. BMC Med. Ethics *18*, 19. https://doi.org/10.1186/s12910-017-0179-8.

55. Chapman, K.R., Tashkin, D.P., and Pye, D.J. (2001). Gender Bias in the Diagnosis of COPD. Chest *119*, 1691–1695. https://doi.org/10.1378/chest.119.6.1691.

56. Sugiyama, M., Krauledat, M., and Müller, K.-R. (2007). Covariate Shift Adaptation by Importance Weighted Cross Validation. J. Mach. Learn. Res. *8*, 985–1005.

57. Zhou, K., Liu, Z., Qiao, Y., Xiang, T., and Loy, C.C. (2022). Domain Generalization: A Survey. IEEE Trans Pattern Anal Mach Intell. *45*, 4396–4415. https://doi.org/10.1109/tpami.2022.3195549.

CellPress
OPEN ACCESS

58. Kouw, W.M., and Loog, M. (2018). An Introduction to Domain Adaptation and Transfer Learning. Preprint at arXiv. https://arxiv.org/abs/1812.11806.

59. Subbaswamy, A., and Saria, S. (2020). From development to deployment: dataset shift, causality, and shift-stable models in health AI. Biostatistics *21*, 345–352. https://doi.org/10.1093/biostatistics/kxz041.

60. Arjovsky, M., Bottou, L., Gulrajani, I., and Lopez-Paz, D. (2019). Invariant Risk Minimization. Preprint at arXiv. https://arxiv.org/abs/1907.02893.

61. Adragna, R., Creager, E., Madras, D., and Zemel, R. (2020). Fairness and Robustness in Invariant Learning: A Case Study in Toxicity Classification. Preprint at arXiv. https://arxiv.org/abs/2011.06485.

62. Hooker, S. (2021). Moving beyond "algorithmic bias is a data problem". Patterns *2*, 100241. https://doi.org/10.1016/j.patter.2021.100241.

About the authors

Eike Petersen holds a PhD in engineering from the University of Lübeck, Germany; an MSc in industrial mathematics from the University of Hamburg, Germany; and a BSc in computer science and engineering from the Hamburg University of Technology, Germany. Currently, he works as a postdoctoral researcher at DTU Compute, Technical University of Denmark, investigating fairness in the context of machine learning in medicine. His research interests lie at the intersection of mathematical modeling, statistical inference, and the responsible application of such techniques in medicine and society.

Sune Holm holds a PhD in philosophy from the University of St. Andrews and is an associate professor at the Department of Food and Resource Economics at the University of Copenhagen. He does research on the ethics of AI with a focus on fairness, explainability, and trustworthiness.

Melanie Ganz is an associate professor at the University of Copenhagen, Denmark. Her PhD in computer science is from the University of Copenhagen, following which she held postdocs at the A.A. Martinos Center for Biomedical Imaging at Massachusetts General Hospital and Harvard Medical School in Boston, Massachusetts, USA, and at the Neurobiology Research Unit at the Copenhagen University Hospital, Denmark. Melanie's research focuses on the application of medical image processing and machine learning to clinical medical image data, mostly neuroscientific data. In addition, she aims to embrace Open Science principles in her research and is hence involved in data sharing initiatives such as the Brain Imaging Data Structure.

Aasa Feragen is a full professor at the Technical University of Denmark. Her PhD in mathematics is from the University of Helsinki, following which she held postdocs at the University of Copenhagen and the MPI for Intelligent Systems in Tübingen. Aasa's research sits at the intersection of machine learning, applied geometry, and medical imaging, where Aasa takes a particular interest in the modeling of data with geometric constraints. Such data include uncertainties and probability distributions, fairness constraints, graphs, trees, and curves. Aasa enjoys contributing to community building, including as program chair of MICCAI 2024, IPMI 2021, and MIDL 2019.

Patterns, Volume *4*

Supplemental information

The path toward equal performance in medical machine learning

Eike Petersen, Sune Holm, Melanie Ganz, and Aasa Feragen

Supplemental note on the bias-variance decomposition provided in the main paper

Our setting in this paper differs from the one in Chen et al.[1] in two ways:

1. We explicitly consider a setting with label errors, i.e., the observed labels Y_{obs} may differ from the true labels Y. As we will see below, this turns out to not affect the decomposition in any way.
2. For simplicity of presentation, we here only consider the case in which the expected mean squared prediction error (or Brier score) is used as the loss function, whereas both Chen et al.[1] and Domingos[2] consider a more general setting with different loss functions. As we point out below, the decomposition provided by Chen et al.[1] fully generalizes to the setting with label errors.

The decomposition we present in the main text follows directly from Theorem 1 in Chen et al.[1]. To see this, note that Chen et al.[1] (and Domingos[2], upon which the derivation in Chen et al. is based) make no assumptions about the *process* by which the model predictions $\hat{y}_D = h_D(x, g)$ are obtained from a training set D. In fact, Chen et al.[1] simply assume that an "algorithm that learns models $\hat{Y}_D$ from datasets D is given, and the covariates X and size of the training data are fixed." They furthermore assume that "$\hat{Y}_D$ is a deterministic function $\hat{y}_D(x, a)$ given the training set D". These assumptions are fully compatible with the case in which the presence of label errors affects the learned model, and the general decomposition provided by theorem 1 in Chen et al.[1] still holds. (In fact, it is not even required that the learning mechanism takes the labels into account at all.) Applying the appropriate simplifications for the case of the squared error loss yields our decomposition. Our decomposition also follows directly from the (simpler, since less general) derivation of the mean-squared error decomposition provided by Bishop[3], by the same argument: the mechanism by which the prediction model is obtained from the training dataset is irrelevant to the derivation. Conditioning on group membership G also does not affect the derivation in any way, since datasets D are drawn independently of G. We discuss in the main text how learning from biased or noisy labels may affect the different terms of the decomposition.

Supplemental References

[1] I. Chen, F. D. Johansson, D. Sontag, Why is my classifier discriminatory?, in: S. Bengio, H. Wallach, H. Larochelle, K. Grauman, N. Cesa-Bianchi, R. Garnett (Eds.), Advances in Neural Information Processing Systems, volume 31, 2018. URL: `https://proceedings.neurips.cc/paper_files/paper/2018/file/1f1baa5b8edac74eb4eaa329f14a0361-Paper.pdf`.

[2] P. Domingos, A unified bias-variance decomposition and its applications, in: Proceedings of the Seventeenth International Conference on Machine Learning, ICML '00, Morgan Kaufmann Publishers Inc., San Francisco, CA, USA, 2000, p. 231–238.

[3] C. M. Bishop, Pattern Recognition and Machine Learning, Information Science and Statistics, Springer New York, NY, 2006.
